# Sudden Cardiac Death due to Ventricular Fibrillation in a Dog Following Packed Red Blood Cell Transfusion

**DOI:** 10.1155/crve/9925419

**Published:** 2025-06-05

**Authors:** Jacob Ryave, Tamilselvam Gunasekaran, Robert A. Sanders

**Affiliations:** Department of Small Animal Clinical Sciences, Michigan State University College of Veterinary Medicine, East Lansing, Michigan, USA

**Keywords:** arrhythmia, canine, Holter

## Abstract

**Objective:** The objective of the study is to report a case of a dog that experienced sudden cardiac death due to ventricular fibrillation following a packed red cell transfusion.

**Case Summary:** A 14-year-old, male castrated, American Staffordshire terrier dog was evaluated before a planned anesthetic event and was diagnosed with second-degree atrioventricular block. One week after the initial evaluation, the dog presented to an emergency facility for episodes of collapse and was diagnosed with hemoperitoneum secondary to splenic rupture. An emergency splenectomy was performed, but the dog developed regenerative anemia postsurgery and received a packed red blood cell transfusion. A Holter monitor was placed before the start of the transfusion due to concerns about previous rhythm abnormalities. Two hours after the blood transfusion, the dog experienced sudden cardiac arrest, and closed-chest cardiopulmonary resuscitation was unsuccessful. Analysis of the Holter recording revealed the onset of ventricular arrhythmias following transfusion that progressed to frequent polymorphic ventricular tachycardia and ventricular fibrillation. Additionally, QT interval prolongation was noted just before the onset of the ventricular arrhythmias on the Holter recording.

**New Information Provided:** To the authors' knowledge, this is the first report of a dog that suffered sudden cardiac death after a blood transfusion and discusses possible causes of ventricular arrhythmias.

## 1. Case Summary

**Figure 1:**
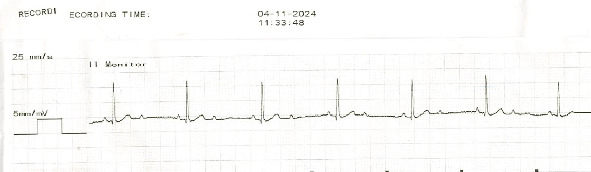
A representative section of the ECG depicting a 2:1 second-degree atrioventricular block in a dog that experienced sudden cardiac death after a red blood cell transfusion. The atrial rate is 104 bpm with a ventricular rate of 60 bpm.

A 14-year-old, 25.4 kg, male castrated, American Staffordshire Terrier dog presented to the cardiology service at Michigan State University Veterinary Medical Center for evaluation of a new-onset heart murmur diagnosed by the referring veterinarian. Physical examination during the referral evaluation confirmed the presence of a Grade II/VI left apical systolic heart murmur with normal synchronous femoral pulses. On transthoracic echocardiography, the dog was diagnosed with myxomatous mitral valve degeneration without any cardiomegaly. Six-lead electrocardiography (ECG) revealed a second-degree atrioventricular (AV) block with 2:1 conduction (atrial rate of 104 bpm) and a ventricular rate of 60 beats per minute (bpm) (Figure 1). The atropine^1^ response test (0.04 mg/kg, IV) revealed an increase in the atrial rate to 170 bpm without a concurrent increase in ventricular rate. At this time, additional diagnostic workup (abdominal ultrasound, tick-borne disease testing, and thoracic radiographs) to assess the cause of second-degree AV block was recommended. Additionally, a permanent, transvenous pacemaker implantation was recommended to manage the bradycardia. However, the owners elected to delay pacemaker implantation until additional diagnostics can be performed with the referring veterinarian.

One week after the initial evaluation, the dog presented to an emergency facility for episodes of tremors, weakness, and collapse. Physical examination on presentation revealed pale pink mucous membranes, prolonged capillary refill time (> 2 s), and discomfort upon abdominal palpation. The dog was noted to have a regular rhythm on cardiac auscultation with heart rate values recorded between 60 and 120 bpm. No ECG was performed at this time. Complete blood count revealed leukocytosis (18.74 K cells/*μ*L, reference interval = 5.2–14.1 K cells/*μ*L), mature neutrophilia (14.74 K cells/*μ*L, reference interval = 3.05–12.1 K cells/*μ*L), regenerative anemia (hematocrit (HCT) 32.1%, reference interval = 35%–60%), total reticulocytes (138.5 K cells/*μ*L, reference interval = 9–115 k cells/*μ*L), and normal thrombocyte count (242 k cells/*μ*L, reference interval = 140–520 k cells/*μ*L). Mild hypokalemia 3.4 mmol/L (3.5–5.0 mmol/L) was noted on blood chemistry. Prothrombin time was within normal limits (16.5 s, reference interval = 12–17 s). Activated partial thromboplastin time was mildly prolonged (105.2 s, reference interval = 71–102 s). Abdominal-focused assessment with sonography for trauma revealed a mild amount of peritoneal effusion, a heterogeneous cavitated mass associated with the head of the spleen. Diagnostic abdominocentesis was not performed. No abnormalities were noted on thoracic radiographs. Abdominal exploratory surgery revealed a ruptured mass associated with the head of the spleen, and total splenectomy was performed. No gross evidence of intra-abdominal metastasis was noted during surgery. The dog remained stable with persistent second-degree AV block during general anesthesia, and no ventricular or atrial ectopy was noted. Following anesthetic recovery, the repeat HCT was 19% with a total protein of 5.8 g/dL (reference interval = 5.6–7.6 g/dL). The dog became intermittently weak and ataxic 8 h postsurgery and collapsed during its walk outside for elimination purposes. A repeat HCT at this time showed no significant changes compared to postsurgery HCT values (20%, reference interval = 36%–60%). Due to the persistence of symptoms, a referral to Michigan State University Veterinary Medical Center was recommended to reassess the cardiac rhythm. However, the clients elected to take the dog home. At home, the dog experienced weakness and collapse while walking outside the following day and was subsequently brought to Michigan State University Veterinary Medical Center emergency and critical care service for further evaluation and continued care.

Upon presentation, the physical examination revealed a bradycardia (heart rate = 55 bpm) consistent with previous examinations. Auscultation revealed a Grade IV/VI left apical systolic murmur with a normal, synchronous femoral pulse. The increase in murmur grade was hypothesized to be secondary to the reduced blood viscosity secondary to significant anemia, though other causes of increased murmur grade could not be fully ruled out. Mucous membranes were pale with normal capillary refill time. Venous blood gas analysis revealed hyperlactatemia (4.7 mmol/L, reference interval = 0.6–3.3 mmol/L) and hypermagnesemia (1.6 mg/dL, reference interval = 0.9–1.4 mg/dL). Complete blood count revealed normocytic, normochromic, regenerative anemia (HCT 23%, total reticulocyte count 27.03 × 10^4^ cells/*μ*L, reference interval = 1.20–7.60×10^4^ cells/*μ*L), leukocytosis (33.9 k/*μ*L, reference interval = 4.6–10.7 k cells/*μ*L), mature neutrophilia (29.8 k cells/*μ*L, reference interval = 2.6–7.5 k cells/*μ*L), monocytosis (1.3 k cells/*μ*L, reference interval = 0.1–0.8 k cells/*μ*L), and mild thrombocytopenia (thrombocytes 110 k cells/*μ*L, reference interval = 140–520 k cells/*μ*L). Polychromasia, Howell–Jolly bodies, and megakaryocytes were present on blood smear evaluation. The chemistry panel revealed hyperchloremia (117 mmol/L, reference interval = 106–115 mmol/L), and hypoproteinemia characterized by hypoalbuminemia (total protein 4.9 g/dL, reference interval = 5.4–6.7 g/dL; albumin 2.2 g/dL, reference interval = 2.8–3.6 g/dL). Urinalysis was unremarkable. A repeat transthoracic echocardiogram revealed no changes to the cardiac size or function despite changes to murmur severity. A repeat ECG revealed a persistent second-degree AV block with 2:1 conduction with a ventricular rate of 58 bpm. Given the stability of the rhythm, the second-degree AV block was not considered to be the isolated cause of the dog's weakness and persistent symptoms. It was hypothesized that the dog's inability to compensate during exercise was due to a moderate level of anemia, which was exacerbated by an underlying second-degree AV block. Therefore, a packed red blood cell (pRBC) transfusion was recommended with concurrent monitoring of the cardiac rhythm. Unfortunately, telemetry or continuous ECG monitoring was not available for real-time monitoring of the cardiac rhythm. Therefore, the dog was fitted with a Holter^2^ monitor to capture cardiac rhythm during any collapse episodes. After blood typing (DEA 1.1+) and cross-matching, the dog received 250 mL of pRBCs over 3 h. The vital parameters remained stable during the initial 2 h of transfusion. However, a rise in the rectal temperature (from 38.2°C to 39.1°C) was noted during the third hour of the transfusion. Based on the volume of pRBCs administered, a 10% increase in HCT was expected posttransfusion. However, the HCT only increased from 22% at baseline to 26% posttransfusion. Additionally, the plasma in the HCT tube was noted to have red discoloration. Two hours posttransfusion, the dog became restless, nauseous, and tachypneic with increased respiratory effort. Ondansetron^3^ (0.5 mg/kg, IV) and diphenhydramine^4^ (2 mg/kg, IM) were administered due to suspicion of an adverse transfusion reaction. The dog arrested soon after the injections, and external cardiopulmonary resuscitation was unsuccessful. A necropsy was not performed.

**Figure 2:**
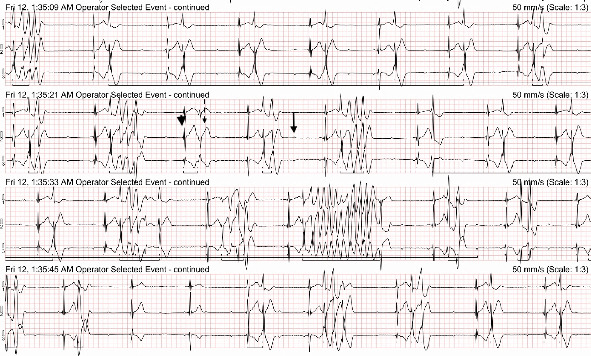
Holter ECG recording depicting second- and third-degree atrioventricular block, junctional escape beats, polymorphic ventricular premature complexes, and runs of ventricular tachycardia. A nonconducted p-wave (black arrow), a junctional escape beat (black arrowhead), and a ventricular premature complex (dotted black arrow) are labeled.

**Figure 3:**
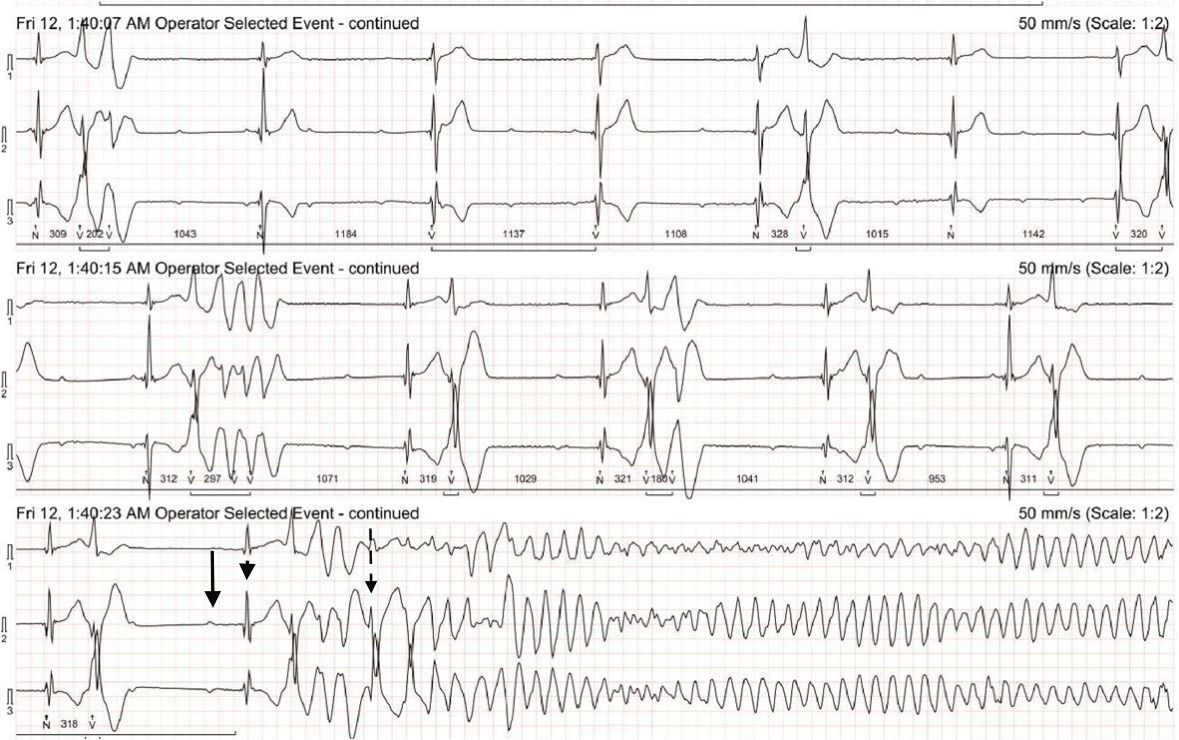
Holter ECG recording showing the onset of ventricular fibrillation (dotted black arrow). A nonconducted p-wave (black arrow) and a junctional escape beat (black arrowhead) are labeled. Frequent polymorphic ventricular premature complexes are visible prior to the onset of ventricular fibrillation.

**Figure 4:**
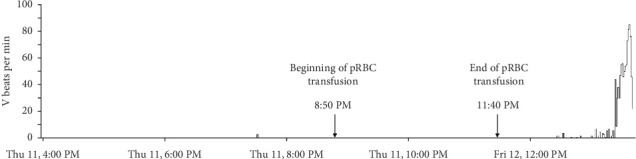
Histogram from Holter recording showing the onset of ventricular beats per minute. Blood transfusion was initiated at 8:50 PM and concluded at 11:40 PM. V: ventricular; pRBC: packed red blood cell.

The Holter recording was analyzed using an automated computer software^5^ with analyses verified by a board-certified cardiologist. Holter recording revealed a persistent 2:1 second-degree AV block before and during the pRBC transfusion. However, 1 h after the pRBC transfusion was completed, a single couplet of ventricular premature complexes (VPCs) was noted. Two hours after the transfusion, the Holter recording revealed polymorphic VPCs with alternating second and third-degree AV blocks with junctional escape beats with the latter (Figure 2). The frequency of the VPCs continued to increase with several couplets, triplets, and short runs of ventricular tachycardia (VT). Ventricular bigeminy was also noted during this time. The morphology of the polymorphic VT episodes was grossly consistent with Torsade de Pointes (TdP) (Figure 3). Four minutes after the onset of the intermittent episodes of VT, the patient experienced ventricular fibrillation (Figure 3) and asystole. The occurrence of VT episodes corresponded to the occurrence of symptoms of restlessness and tachypnea in this dog. A histogram of total ventricular beats showed that the ventricular arrhythmias only occurred after pRBC transfusion (Figure 4). The QT interval was measured for 10 sinus beats during each hour of the Holter recording (Table 1) [1]. The corrected QT interval was calculated using the Fridericia method (corrected QT = QT/∛RR, where RR is the preceding R-to-R interval). Interestingly, prolongation of the corrected QT interval was noted 1 h before the onset of VF (Table 1).

## 2. Discussion

A notable aspect of this case report is that the ventricular arrhythmias only became apparent on the Holter recording (Figure 4) approximately 1 h after the completion of the pRBC transfusion. The occurrence of ventricular arrhythmias also coincided with the onset of QT interval prolongation on the Holter recording. This timing suggests that acute changes related to pRBC transfusion could have contributed to the onset of ventricular arrhythmias in this dog. More specifically, acquired QT interval prolongation due to citrate toxicity, acute transfusion reactions, and hypoxic myocardial injury are considered primary possibilities. Acquired long QT syndrome is a disorder of cardiac repolarization most often caused by specific drugs, hypokalemia, hypocalcemia, or hypomagnesemia resulting in polymorphic VT and sudden cardiac death [2]. Acquired QT interval prolongation and the subsequent development of TdP following blood transfusions have been documented in humans [3–5]. In such cases, chelation of ionized calcium and magnesium by sodium citrate in the blood products and resulting hypocalcemia and hypomagnesemia were considered to cause QT interval prolongation [4–6]. Most reports of such citrate toxicity and resulting electrolyte derangements are associated with massive blood transfusions in human patients [7]. One report in dogs documented ionized hypocalcemia and hypomagnesemia in dogs receiving pRBC transfusion of more than 22.5 mL/kg [8]. The dog in this report did not receive a massive pRBC transfusion (received only 10 mL/kg). However, in humans, the dose of administered blood transfusion has been directly correlated to the degree of ionized hypocalcemia with some patients developing hypocalcemia even after only receiving one unit of blood products [5, 6]. Unfortunately, serum electrolytes were not measured during or after the pRBC transfusion in this dog, making it challenging to draw definitive conclusions on any electrolyte derangements contributing to QT interval prolongation. Additionally, this dog received intravenous ondansetron immediately before cardiac arrest, a drug known to cause QT interval prolongation [9–11]. However, the dog was noted to have ventricular arrhythmias and QT interval prolongation even before ondansetron administration. It is possible that intravenous ondansetron played an additive role in causing acute exacerbation of the QT interval prolongation and VF initiation.

The clinical findings of hyperthermia during pRBC transfusion, less than expected increase in HCT after transfusion, and the appearance of a hemolyzed plasma in the HCT tube raise a possibility of acute–hemolytic transfusion reaction in this dog [12]. An immunologic, acute hemolytic reaction is less likely, given no prior history of blood transfusions and normal cross-matching before pRBC administration [12]. However, nonimmunologic hemolytic transfusion reactions that occur due to thermal, osmotic, mechanical, or chemical factors that damage transfused blood cells resulting in acute or delayed hemolysis should be considered [13, 14]. Acute hemolytic reaction and resulting hypoxic injury could also trigger ventricular arrhythmias and VF [2]. Finally, the impact of this dog's underlying second-degree AV block as a trigger for VF should be considered. Sudden cardiac death has been reported in dogs with high-grade second-degree and third-degree AV block [15]. In any bradycardic rhythm with a long baseline ventricular cycle length, a sudden prolongation in cycle length can trigger arrhythmogenic early after depolarizations and TdP [16]. However, the Holter recording revealed no ventricular arrhythmias before the pRBC transfusion, with VPCs only appearing after the transfusion was completed. This timing suggests that the underlying second-degree AV block is less likely to be the sole cause of TdP in this dog. Nevertheless, any factor that prolongs the QT interval could potentially amplify the risk of ventricular fibrillation in the presence of the dog's second-degree AV block.

To the authors' knowledge, this is the first report documenting sudden cardiac death due to VF following a blood transfusion in a dog. While the exact cause of sudden cardiac death remains unknown in this dog, a multitude of factors including possible electrolyte abnormalities related to pRBC transfusion, acute transfusion reactions, and the second-degree AV block could have played a role in QT interval prolongation and precipitation of VF this dog. This case report highlights the need for monitoring electrolytes, ionized calcium levels, and potentially continuous ECG monitoring in dogs during and after blood transfusion, especially in dogs with underlying cardiac rhythm disturbances.

## Figures and Tables

**Table 1:** QT interval measurements in a dog that experienced sudden cardiac death after packed red blood cell transfusion.

**Time**	**QTcF mean (seconds)**
4 PM	0.296
5 PM	0.299
6 PM	0.297
7 PM	0.298
8 PM	0.295
9 PM	0.301
10 PM	0.288
11 PM	0.270
12 AM	0.291
**1 AM**	**0.324**

*Note:* Corrected QT interval calculated using Fridericia's method for 10 consecutive sinus beats during the corresponding hour of the Holter monitoring period. The blood transfusion began at 8:50 PM and concluded at 11:40 PM. The onset of ventricular arrhythmias occurred shortly after 1:20 AM. QTcF: corrected QT interval using Fridericia's method [1]. The bolded line (1 AM, 0.324) was intended to highlight the increase in QTcF at that hour compared to the previous time points.

## Data Availability

Data sharing is not applicable to this article as no datasets were generated or analyzed during the current study.

## Nomenclature

bpm: beats per minute
ECG: electrocardiography
HCT: hematocrit
pRBC: packed red blood cell
TdP: Torsades de Pointes
VF: ventricular fibrillation
VPC: ventricular premature complex

## Endnotes

^1^Atropine, Vedco Inc., St. Joseph, Missouri

^2^Lifecard CF, Spacelabs Healthcare, Snoqualmie, Wisconsin, United States

^3^Ondansetron, Accord Healthcare Inc., Durham, NC

^4^Diphenhydramine, Bionechepharma, Lake Forest, IL

^5^Pathfinder SL, Spacelabs Healthcare, Snoqualmie, Wisconsin, United States
